# Amnion formation in the mouse embryo: the single amniochorionic fold model

**DOI:** 10.1186/1471-213X-11-48

**Published:** 2011-08-01

**Authors:** Paulo NG Pereira, Mariya P Dobreva, Liz Graham, Danny Huylebroeck, Kirstie A Lawson, AN Zwijsen

**Affiliations:** 1 Laboratory of Developmental Signaling of the Department of Molecular and Developmental Genetics (VIB11), VIB, Leuven, Belgium; 2 Laboratory of Molecular Biology (Celgen) of the Department of Molecular and Developmental Genetics (VIB11), VIB, Leuven, Belgium; 3 Center for Human Genetics, K.U. Leuven, Leuven, Belgium; 4 MRC Human Genetics Unit, Institute of Genetics and Molecular Medicine, Western General Hospital, Edinburgh, UK

**Keywords:** allantois, amniochorionic fold, amniotic membrane, anterior separation point, apoptosis, bone morphogenetic proteins, chorion, epiblast, gastrulation

## Abstract

**Background:**

Despite the detailed knowledge obtained over the last decade on the molecular regulation of gastrulation in amniotes, the process of amnion development has been poorly described and illustrated in mice, and conflicting descriptions exist. Understanding the morphogenesis and development not only of the early mouse embryo, but also of its extraembryonic tissues, is crucial for correctly interpreting fate-mapping data and mouse mutants with gastrulation defects. Moreover, the recent isolation from amnion of cells with stem cell features further argues for a better understanding of the process of amnion formation. Here, we revisit the highly dynamic process of amnion formation in the mouse. Amnion development starts early during gastrulation and is intimately related to the formation of the exocoelom and the expansion of the amniotic fold. The authoritative description involves the fusion of two amniotic folds, a big posterior and a smaller anterior fold. We challenged this 'two amniotic folds' model by performing detailed histomorphological analyses of dissected, staged embryos and 3D reconstructions using historical sections.

**Results:**

A posterior fold of extraembryonic ectoderm and associated epiblast is formed early during gastrulation by accumulation of extraembryonic mesoderm posterior to the primitive streak. Previously called the "posterior amniotic fold", we rename it the "amniochorionic fold" (ACF) because it forms both amnion and chorion. Exocoelom formation within the ACF seems not to involve apoptosis within the mesoderm. The ACF and exocoelom expand without disrupting the anterior junction of epiblast, extraembryonic ectoderm and visceral endoderm. No separate anterior fold is formed; its absence was confirmed in 3D reconstructions. Amnion and chorion closure is eccentric, close to the anterior margin of the egg cylinder: we name it the "anterior separation point".

**Conclusions:**

Here, we reconcile previous descriptions of amnion formation and provide new nomenclature, as well as an animation, that clarify and emphasize the arrangement of the tissues that contribute to amnion development and the dynamics of the process. According to our data, the amnion and the chorion are formed by a single amniochorionic fold initiated posteriorly. Finally, we give an overview on mutant mouse models with impaired amnion development.

## Background

**Figure 1:**
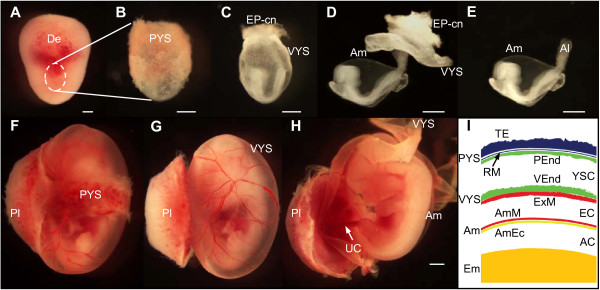
**Extraembryonic tissues and organs in a mouse embryo and foetus**. **(A-E) **Progressive dissection of an E8.5 mouse conceptus revealing its extraembryonic tissues. **(A) **Deciduum (De) as isolated from the uterus. **(B) **Isolated conceptus: only the parietal yolk sac (PYS), including Reichert's membrane is visible. **(C) **Upon removal of the PYS the ectoplacental cone (EPCn), visceral yolk sac (VYS) and embryo proper become visible. **(D-E) **When the VYS is, subsequently, removed the amnion (Am), the allantois (Al) and the embryo proper become better visible. Scale bar: 500 μm **(F-H) **E12.5 mouse conceptus. **(F) **Foetus within its PYS and VYS, with intact placenta (Pl). **(G) **Removal of the PYS reveals the vascularized VYS. **(H) **Avascular amnion and the umbilical cord (UC) are visible when the foetus is dissected free from VYS. Scale bar: 1 mm **(I) **Schematic representation of the extraembryonic tissues at the level of the dashed line in (F), and their composition. Additional abbreviations in the scheme: AmEc: amniotic ectoderm; AmM: amniotic mesoderm; AC: amniotic cavity; EC: exocoelomic cavity; Em: embryo; ExM: extraembryonic mesoderm; PEnd: parietal endoderm; RM: Reichert's membrane; TE: trophectoderm; VEnd: visceral endoderm; YSC: yolk sac cavity.

To develop and survive *in utero*, the mammalian conceptus develops a number of extraembryonic tissues and organs to provide nutritional support and protection to the embryo proper. These extraembryonic appendages are shed at birth. The amnion is the innermost extraembryonic membrane that surrounds the foetus of amniotes and delineates the fluid-filled amniotic cavity, thereby providing a confined niche within the conceptus and conferring protection and shock resistance [1]. Of all the extraembryonic membranes it is morphologically the most conserved membrane. In contrast to the visceral yolk sac, the chorion and the allantois, the amnion is a thin transparent membrane (Figure 1E, H) that is avascular in most amniotes. In mouse embryos, the amnion consists throughout gestation of a bi-layered membrane of squamous mesoderm and ectoderm facing the exocoelomic and the amniotic cavity respectively [2,3] (Figure 1I). The amniotic ectoderm is continuous with the embryonic ectoderm, whereas the amniotic mesoderm shares its borders with the mesothelium of the visceral yolk sac and the allantois. Progressively, a basal lamina composed of collagen, laminin, nidogen and fibronectin fibers forms between the amniotic ectoderm and mesoderm [4-6]. The amniotic epithelium acquires an increasing number of microvilli at the surface, which may be associated with enhanced filtering and transport capacity across the membrane [7].

Due to their lordotic position, mouse and rat embryos are peculiar in possessing inverted germ layers in which the ectoderm initially faces the inside of the egg cylinder [3]. Starting at the 9-10-somite stage, mouse embryos undergo axial rotation, and hence achieve the regular flexed foetal position. Consequently, the anterior junction between the embryo proper and the amnion on the one hand, and the embryo and the yolk sac on the other hand shifts progressively from anterior ectoderm over the heart field, to ventrally where the vitelline vein contacts the body wall [8]. When turning is finished (14-16 somite stage), the embryo has become entirely enfolded in the amnion and visceral yolk sac [3,9] (Figure 1G).

The amniotic membrane has low immunogenicity and hence high potential for regenerative medicine [10,11]. Indeed, the amnion has been used for a century as a wound dressing [12]. Recently, the amnion has gained attention due to the apparent presence of resident stem cells in term human amniotic ectoderm [13]. Furthermore, cells isolated from human term amniotic ectoderm and mesoderm showed triple lineage differentiation capacity in cell culture [14-17]. Similar studies on rat and mouse amniotic-membrane-derived cells have reported the existence of such pluripotent cells [18,19]. The origin of the amniotic stem cells is, however, unclear and, in the case of mice and rats, the source used to isolate the so-called amniotic stem cells has sometimes been controversial [9].

Amnion formation is intimately related to the formation of the primitive streak early during gastrulation, but most investigators have focused on the analysis of the embryonic component of the conceptus, typically discarding the amnion in their studies. Hence, the developmental origin of mouse amnion and its formation have been described fragmentarily [3,20,21]. The process of dividing the proamniotic cavity into the exocoelomic and the amniotic cavity by a membrane called the amnion is completed at the late streak/early bud to neural plate stage, depending on the mouse strain investigated.

The primitive streak is the first morphological landmark of gastrulation at late TS 9 (E6.5). It is characterized by a thickening at the posterior side of the epiblast, close to the embryonic-extraembryonic junction. Streak ectoderm undergoes an epithelial-to-mesenchymal transition, and mesoderm emerges [22-25]. Cells from the epiblast, via the streak, give rise to the mesoderm, including embryonic mesoderm and extraembryonic mesoderm of the chorion, amnion, yolk sac and allantois [26]. Moreover, the epiblast gives rise to amniotic ectoderm as well as to embryonic ectoderm, endoderm and primordial germ cells [2]. The extraembryonic ectoderm will form the chorion and, with the ectoplacental cone, the chorionic disk of the placenta. Visceral endoderm (VEnd) becomes the endoderm component of the visceral yolk sac.

Fate mapping studies have revealed that amniotic mesoderm and amniotic ectoderm are derived from different regions of the epiblast. Descendants of epiblast cells located at the posterior and posterolateral sides of the epiblast contribute to amniotic mesoderm [25]. Indeed, labelling cells of the posterior primitive streak showed that the mesoderm derivative is mostly extraembryonic, part of which contributes to the formation of the amnion during early gastrulation (early- and midstreak) [27,28]. In contrast proximal epiblast that is in the anterior half of the embryo at prestreak and streak stages, gives rise to amniotic ectoderm [25].

Amnion formation begins with the accumulation of extraembryonic mesoderm leading to the formation of a posterior amniotic fold [3,21,29,30] followed by folds along the sides of the egg cylinder like the progression of the lateral mesoderm wings [21]. Bonnevie (1950) disputed the role and existence of a posterior fold, but highlighted that the extraembryonic ectoderm at the anterior margin of the egg cylinder remains closely associated with the visceral endoderm, despite the eventual intercalation by extraembryonic mesoderm. Snell & Stevens (1966) emphasized that extraembryonic mesoderm may accumulate at the anterior margin and regarded it as a small anterior fold. The exocoelomic cavity is then formed by the accumulation and coalescence of "*small cavities*", or "*small closed lumina" *[20], within the posterior and lateral folds. According to Snell & Stevens (1966), the posterior, lateral and anterior folds should, however, be thought of as a continuous constriction around the middle of the egg cylinder that tightens as the folds develop. This description differs from Kaufman's authoritative description (1992), which proposes the existence of separate posterior and anterior amniotic folds, each with an exocoelomic cavity. Kaufman (1992) described and illustrated the subsequent amnion expansion as follows: "*The rapid expansion of the posterior amniotic fold and its apposition and eventual fusion with the considerably smaller anterior amniotic fold results in the formation of the chorion and amnion, which divide the proamniotic cavity into ectoplacental, exocoelomic and amniotic cavities, respectively" *(plate 5 in *The Atlas of Mouse Development *[3]). Several phenotypes observed in the amnion of mutant mouse models have been interpreted according to Kaufman's description [31-34]. However, during routine analysis of serial sections, we came to the conclusion that this description might be inaccurate because we never observed an anterior amniotic fold with exocoelom. Therefore, we re-examined the process of amnion formation in the mouse based on histological analysis of mouse embryos between the prestreak (E6.0) and the neural plate stage (E7.5). Computer reconstruction of histological sections used for *The Atlas of Mouse Development *confirmed the absence of an anterior fold. Finally, we provide an animation that illustrates the single amniochorionic fold model and emphasizes the dynamics and arrangement of the tissues that contribute to amnion development.

## Results

**Figure 2:**
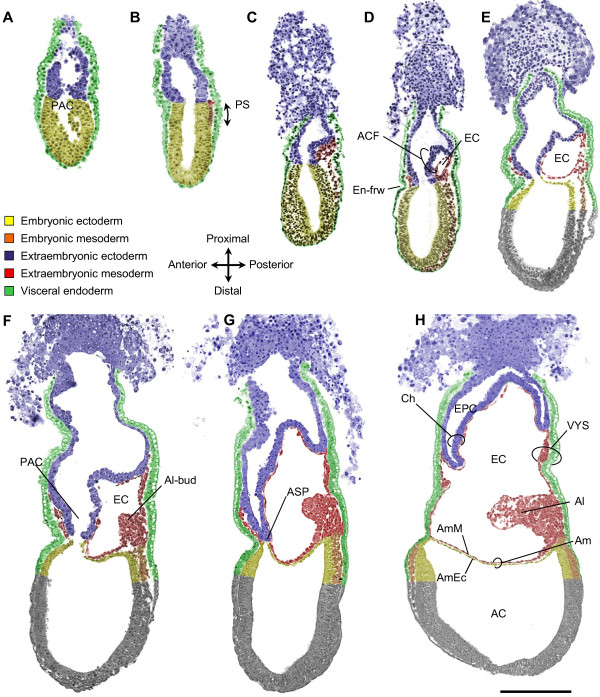
**Amnion formation in mouse embryos, illustrated by longitudinal sections**. Sections at the midline in the extraembryonic-embryonic region of embryos between E6.0 and E7.5 illustrate the different stages of amnion development. The tissue layers in the extraembryonic and extraembryonic-embryonic junction region were artificially painted based on histomorphological differences and the analysis of layer-specific markers (Figure 4). **(A) ***Prestreak stage: *no mesoderm, no amniochorionic fold (ACF). **(B) ***Early streak stage: *extraembryonic and embryonic mesoderm emerges at the primitive streak (PS). **(C) ***Midstreak stage*: extracellular spaces accumulate within the extraembryonic mesoderm. **(D) ***Late streak/no bud stage*: the ACF protrudes into the proamniotic cavity (PAC) and coalescence of spaces in the extraembryonic mesoderm generates the exocoelomic cavity (EC). The endodermal furrow (En-frw) marks the anterior midline at the extraembryonic-embryonic junction. **(E) **Between *late streak/no bud *and *late streak/early bud stage*: expansion of the EC. **(F) ***Late streak/early bud stage: *a large ACF extends from the posterior. The allantoic bud (Al-bud) has become visible. **(G) **Between *late streak/early bud *and *neural plate stage: *the lateral extensions of the EC meet at the focal anterior separation point (ASP). Closure and separation of the ectodermal lineages occurs. **(H) ***Neural plate stage: *the segregated amniotic (Am) and chorionic (Ch) membranes divide the PAC into the amniotic cavity (AC), EC and ectoplacental cavity (EPC). The allantois (Al) and visceral yolk sac (VYS) become clear. The amniotic ectoderm (AmEc) and mesoderm (AmM) acquire their squamous architecture. Sections do not go through the midline at the posterior (E) or anterior (H) side at the extraembryonic-embryonic junction of the conceptus. Scale bar: 200 μm.

**Figure 3:**
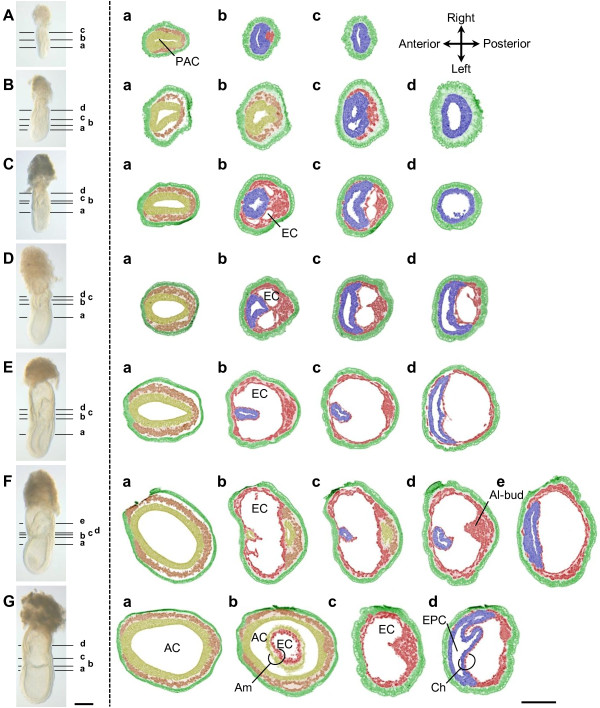
**Amnion Formation In Mouse Embryos, Illustrated By Transverse Sections**. Transverse sections of embryos between E6.5 and E7.5 illustrate the different stages of amnion development. Each section plane is represented by a corresponding line in the picture of a matching whole-mount embryo. Artificial colors as in Figure 2. No attempt has been made to distinguish the head process and any definitive endoderm from the embryonic mesoderm and visceral endoderm, respectively. **(A) ***Early streak stage: *extraembryonic mesoderm has inserted between extraembryonic ectoderm and visceral endoderm at the posterior side of the embryo. **(B) ***Midstreak stage*: extracellular spaces are present within the extraembryonic mesoderm. **(C) ***Late streak/no bud stage*: lacunae in extraembryonic mesoderm coalesce to form the exocoelomic cavity (EC). **(D) **Between *late streak/no bud *and *late streak/early bud stage*: the EC has enlarged. **(E) ***Late streak/early bud stage: *the EC extends laterally around the egg cylinder, converging on the anterior midline at the embryonic-extraembryonic junction. **(F) **Between *late streak/early bud *and *neural plate stage: *the proamniotic cavity (PAC) is constricted by the embryonic ectoderm and extraembryonic ectoderm of the amniochorionic fold, preceding the closure and separation of amnion and chorion. The allantoic bud (Al-bud) is prominent. **(G) ***Neural plate stage: *the amnion (Am) and chorion (Ch) are completely segregated dividing the proamniotic cavity into the amniotic cavity (AC), exocoelomic cavity and ectoplacental cavity (EPC). Scale bar: 200 μm.

**Figure 4:**
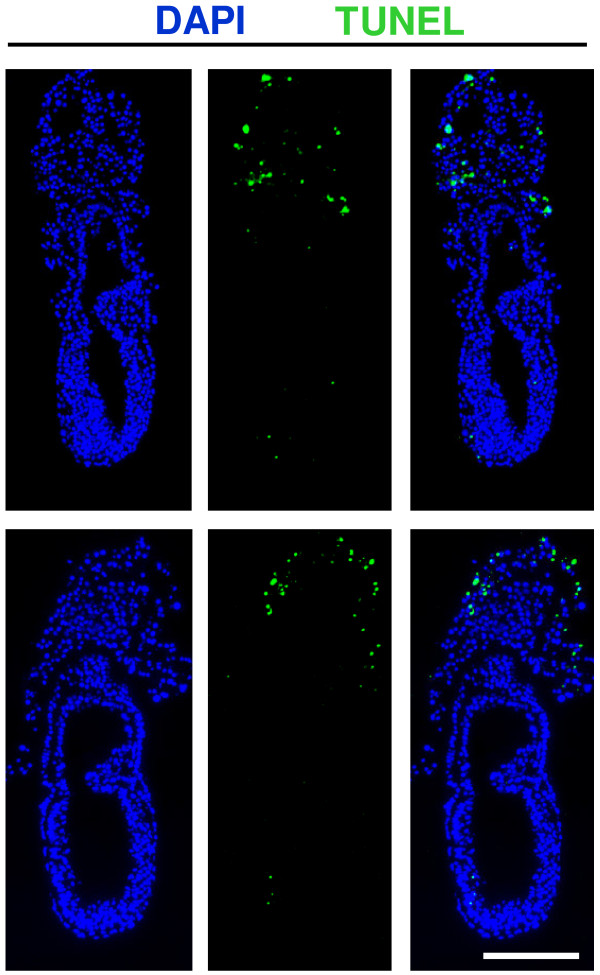
**Absence of apoptosis in the amniochorionic fold of embryos undergoing exocoelom formation**. **(A-B) **TUNEL assay in sections of embryos showing absence of programmed cell death in extraembryonic mesoderm during the process of exocoelom formation. Apoptotic cells were readily detected in the ectoplacental cone. Scale bar: 200 μm.

Amnion formation begins early during gastrulation in the mouse (Figure 2). At the midstreak stage, extraembryonic mesoderm accumulates between extraembryonic ectoderm and visceral endoderm at the posterior side of the embryo and a fold of extraembryonic and embryonic ectoderm bulges into the proamniotic cavity (Figure 2B, C; see also Figure 3A). This fold was historically called the "posterior amniotic fold", but we propose that it be named *amniochorionic fold *(ACF) because it gives rise to both the amnion and chorion. As the fold starts to form, the proamniotic canal becomes eccentric. Subsequently, small lacunae appear within the extraembryonic mesoderm (Figure 2C; see also Figure 3B): the mechanism of lacunae formation is, however, unknown. It was possible that exocoelom formation could involve programmed cell death, similar to cavitation in the epiblast that leads to proamniotic cavity formation [35]. However, we did not detect apoptosis in posterior extraembryonic mesoderm during the process of exocoelom formation (n = 5, Figure 4), making it likely that another process is involved. Whatever the mechanism, the lacunae accumulate and fuse readily to form a larger cavity, the exocoelom, which is characteristically seen in late streak stage embryos (Figure 2D; see also Figure 3C, D).

**Figure 5:**
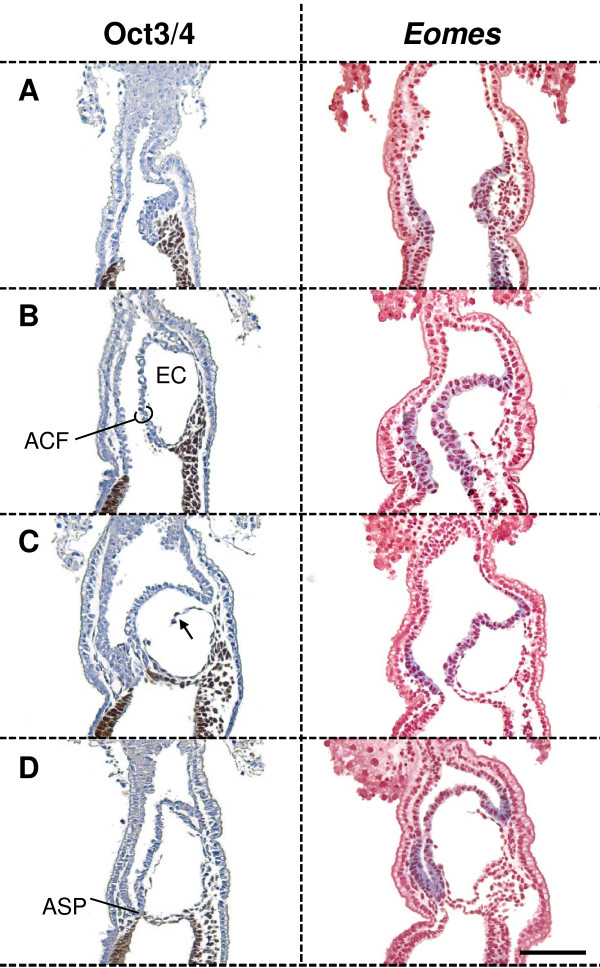
**Embryonic and extraembryonic ectoderm demarcation in the amniochorionic fold**. Embryonic ectoderm was marked by IHC for Oct3/4 (brown), while extraembryonic ectoderm was marked by the expression of *Eomes *(blue). The latter is also detected in nascent embryonic and extraembryonic mesoderm. **(A) **Prior to the formation of the exocoelomic cavity (EC), the amniochorionic fold (ACF) is recognized by the extraembryonic mesoderm accumulation between extraembryonic ectoderm and visceral endoderm. **(B) **Soon after formation of the EC, the extraembryonic ectoderm is the major contributor for the ectoderm of the ACF. **(C) **The contribution of embryonic ectoderm to the ACF increases progressively as the embryo continues to grow, and the EC enlarges. The black arrow indicates the transient non-alignment that often appears between the mesoderm and extraembryonic ectoderm of the ACF. **(D) **At the anterior separation point (ASP), embryonic ectoderm is aligned with extraembryonic mesoderm in the lower half of the ACF to form the amnion, while the extraembryonic ectoderm apposed to extraembryonic mesoderm in the upper half of the fold forms the chorion. Scale bar: 200 μm.

So far our observations fitted with the descriptions by Kaufman and others in the field. The amniochorionic fold, which delineates the exocoelom, consists of extraembryonic mesoderm facing the exocoelom, and of a sheet of ectoderm facing the proamniotic cavity. However, often the ectoderm and mesoderm of the fold are transiently not fully aligned (Figure 5C arrow) [21]. The ectoderm of the ACF has an epiblast-derived component and an extraembryonic-ectoderm-derived one, as shown by the presence of Oct3/4 and expression of *Eomes *in the respective layers (Figure 5) [36,37]. The epiblast-derived component and the extraembryonic-ectoderm component of the ACF form the prospective amniotic ectoderm and chorionic ectoderm, respectively. At the midstreak stage, the ectoderm of the ACF consists mostly of extraembryonic ectoderm (Figure 5A, B). However, as the fold enlarges, the contribution of embryonic ectoderm to the ACF gradually increases (Figure 5C, D).

**Figure 6:**
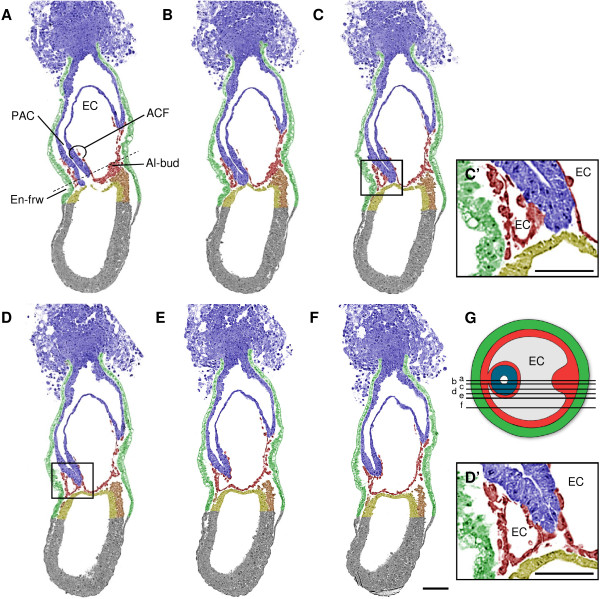
**Series of longitudinal sections of an embryo with large exocoelomic cavity (ec)**. Artificial colors as in Figure 2. **(A) **Midline section characterized by the presence of the allantoic bud (Al-bud), and endodermal furrow (En-frw) close to the prospective anterior separation point. **(B) **Section adjacent to the midline section A. **(C-D) **Neighboring sections cut twice through the amniochorionic fold (ACF), which result *seemingly *in an anterior fold and posterior fold with their respective cavities. **(E) **A more lateral section reveals that the exocoelomic cavity extends around the egg cylinder. **(F) **Most lateral section of the series. Scale bar: 100 μm **(G) **Position of the longitudinal sections (A to F) on a schematic transverse section of the embryo at the level of the dashed line in A. Grey reflects the EC; no fill represents the proamniotic canal (PAC). **(C'-D') **Magnifications of the boxed areas shown in figure panel C and D, respectively. Scale bar: 50 μm.

In contrast to what has been described by Kaufman (1992), we did not find evidence for a separate anterior amniotic fold with its own exocoelomic cavity. Instead, a single cavity - the exocoelomic cavity - extends laterally around the egg cylinder as wing-like lateral extensions (Figure 2E, F); this process was further confirmed by transverse sections (Figure 3D, E). The lateral extensions converge on the anterior midline at the embryonic-extraembryonic junction (Figure 2F). On a parasagittal section of a late streak embryo, halfway between the midline and the lateral side of the egg cylinder (Figure 6C, D), there appear to be two exocoeloms, a large posterior one and a smaller anterior one, which could fit with Kaufman's interpretation. However, analysis of the neighbouring serial sections (Figure 6E, F) reveals that this "anterior cavity" is actually continuous with the "posterior cavity", as represented in the scheme (Figure 6G). Hence, there is no anterior cavity in a midline section in the embryonic-extraembryonic junction region, when the allantoic bud and anterior endodermal furrow are in the same plane (Figure 2E, F; Figure 3D, E; Figure 6A). Therefore, we conclude that both "cavities" are the result of a cut through one single fold and the exocoelomic cavity.

The proamniotic canal becomes localized anteriorly (Figure 2F; Figure 3E), close to where the lateral wings of the exocoelom converge. Here, the extraembryonic and embryonic ectoderm from the ACF will contact their counterparts at the anterior side of the egg cylinder resulting in the closure of the amniotic cavity and the separation of embryonic and extraembryonic ectoderm. We propose to name the latter region the *anterior separation point *(ASP) (Figure 2G; Figure 3F). The ectoderm of the embryo proper and the amniotic ectoderm now delineate the amniotic cavity completely, and the extraembryonic ectoderm is now called chorionic ectoderm. The junction between presumptive chorionic ectoderm and amniotic ectoderm remains distinct, without apparent cell mingling across the visible anatomical junction between extraembryonic ectoderm and embryonic ectoderm in the amniochorionic fold, as shown by the complementary expression patterns of *Eomes *(extraembryonic ectoderm) and presence of Oct3/4 (ectoderm layer of the amnion) (Figure 5D).

**Figure 7:**
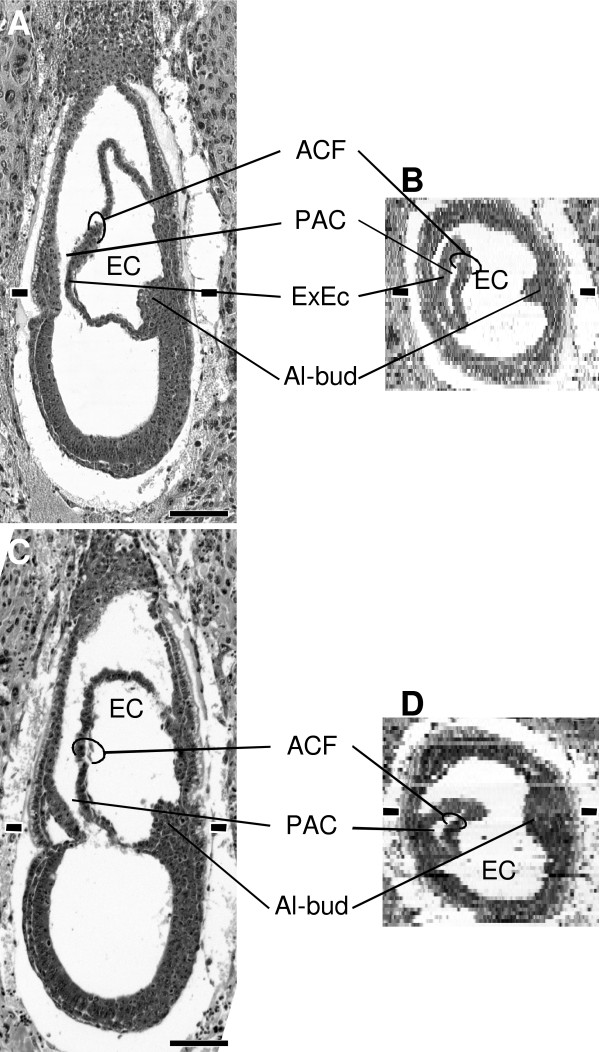
**Reconstruction of embryos prepared for kaufman's *the atlas of mouse development***. **(A) **Midline longitudinal section from the embryo displayed in plate 5 a-e of *The Atlas of Mouse Development*. Scale bar: 100 μm **(B) **Transverse slice at the level of the dashes in A after 3D computer reconstruction of the consecutive sections of the embryo used for plate 5 a-e. The pixelation is mainly due to the thickness of the sections (7 μm), compared with the 0.34 μm resolution in A. **(C) **Longitudinal section of another embryo from Kaufman's collection. This embryo was not included in *The Atlas of Mouse Development*. **(D) **Transverse slice at the level of the dashes in C after 3D computer reconstruction of the consecutive sections of the embryo. Dashes in D indicates the level of the section shown in C. Abbreviations: ACF: amniochorionic fold; Albud: allantoic bud; EC: exocoelomic cavity; ExEc: extraembryonic ectoderm; PAC: proamniotic cavity.

Although the amniotic and chorionic ectoderm are now separated, the mesoderm of the fold that will intercalate between both ectoderm layers is not yet physically divided into chorionic and amniotic mesoderm. The exocoelomic cavity continues to enlarge with accumulation of extraembryonic mesoderm to form visceral yolk sac, allantois and blood islands further segregating the amnion and the chorion (Figure 2H). The exocoelomic cavity is now delineated all round by extraembryonic mesoderm of the visceral yolk sac (Figure 2H; Figure 3G). As a consequence of the membrane segregation, the chorionic mesoderm and amniotic mesoderm become apposed to extraembryonic and epiblast-derived ectoderm, respectively. The amniotic and chorionic membranes then divide the proamniotic cavity of the egg cylinder into the amniotic, exocoelomic and ectoplacental cavities (Figure 2H). In some mouse strains, the allantoic bud - the precursor of the allantois - is already visible before amnion closure [3] (Figure 2F), whereas it may appear after closure in other strains [38]. To challenge the single amniochorionic fold model further, also in an independent genetic background, we re-examined the original serial, longitudinal sections of two embryos that were prepared for *The Atlas of Mouse Development *[3], and generated 3D computer reconstructions thereof. An independent anterior amniotic fold was not found in transverse slices extracted from the 3D reconstructions (Figure 7B, D). Indeed, the reconstructions of these historical reference sections confirmed the presence of a single exocoelom extending around the egg cylinder (Figure 7).

Since exocoelom formation and its consequence for amnion and chorion formation are highly dynamic processes which are difficult to envision, we clarify the process in an animation (Additional file 1).

## Discussion

Earlier descriptions of amnion and exocoelom formation in the mouse have been partial and conflicting. Both Snell & Stevens (1966) and Kaufman (1992) describe a small *anterior amniotic fold*. However, Snell & Stevens consider this anterior fold a *continuation *of the posterior and lateral amniotic folds, and they do not describe it having lacunae. Conversely, Kaufman describes an anterior fold with an independent exocoelom. Our present data demonstrates the absence of an independent, exocoelom containing, anterior fold. On the other hand, we show the presence of a single fold that is initiated posteriorly, and which we redefine as the ***amniochorionic fold (ACF)***. The fold expands laterally around the egg cylinder, like the progression of the lateral mesodermal wings. The lateral extensions converge on the anterior midline. The expansion of the exocoelomic cavity of the ACF accompanies the lateral expansion of the fold around the egg cylinder, but does not reach the anterior side of the embryo. Instead, a local accumulation of mesoderm can occur, forming what could be interpreted as a small anterior fold (Figure 6A; Figure 7A). We, however, propose not to call this small bulge an anterior fold because it risks being confusing. Interestingly, while the epiblast grows directionally towards the primitive streak [25,39], the proamniotic canal remains localized anteriorly, close to where the exocoelom wings converge, and maintains a relatively constant diameter before closure at the level of the embryonic-extraembryonic junction (Figure 3C-3E). This may promote the formation and expansion of the exocoelomic cavity within the extraembryonic mesoderm. Compared with the growing embryo, relatively little cellular material is required in the developing amnion, chorion and yolk sac by virtue of exocoelom formation. Ultimately, amnion closure is eccentric, close to the anterior margin of the egg cylinder, which we define as the ***anterior separation point (ASP)***.

The differences in interpretation of amnion formation may be partly explained by the difficulties in correctly orienting and staging mouse embryos when sectioned within the deciduum, but also to slight variations in the expansion of the exocoelom on the left and right sides and to residual adjustment of axial symmetry of the embryo [40,41]. In our study, we analysed *whole-mount *embryos dissected free from the deciduum to better control the plane of section at the embryonic-extraembryonic junction. For instance, to visualize the ASP in a midline section, it is crucial to examine a section in which the endodermal furrow and the base of the allantois are both present.

We propose a model of amnion formation in the mouse involving a single ACF growing and expanding laterally from the posterior side of the embryo: **the single amniochorionic fold model**. The 3D-reconstructions of Kaufman's (1992) original serial sections support our model further. The new material was in a CD1 background (Figure 2 to Figure 6), and the model was confirmed in an F1 (C57B6xCBA) background (Figure 7). However strain dependent differences in the formation of the amnion cannot be fully excluded.

Amnion development in the mouse is intimately related to exocoelom expansion. The initial establishment of the exocoelom is intriguing. The question remains as to what cellular and molecular mechanisms drive the formation of the lacunae in the extraembryonic mesoderm. Selective cell survival and programmed cell death have been implicated in causing the cavitation in epiblast leading to the formation of the proamniotic cavity [35]. Should a similar mechanism drive the formation of the exocoelomic cavity, cells at multiple sites throughout the extraembryonic mesoderm would have to undergo programmed cell death to generate the scattered small individual cavities. However, we did not detect apoptosis in the mesoderm of the fold, indicating that programmed cell death is likely not involved in the process of exocoelom formation. Perhaps the formation of the exocoelomic cavity reflects merely the enlargement of extracellular spaces, or depends on the continuous rearrangement of cell adhesion molecules and extracellular matrix, allowing the formation of spaces in-between the mesodermal cells of the ACF, similar to vascular lumen formation in invertebrates and vertebrates [42,43]. The accumulation and coalescence of these extracellular spaces or lacunae leads to the formation of a large extraembryonic coelom - the exocoelom - lined by extraembryonic mesoderm. To our knowledge, there are no mutants reported with explicitly impaired exocoelom formation in the newly formed extraembryonic mesoderm. Nevertheless, the ectopic appearance of the cell adhesion molecule VCAM and its receptor α1-integrin on the extraembryonic mesoderm lining the exocoelom (amniotic, chorionic and yolk sac mesoderm component) in FoxF1-deficient mice leads to a compressed/ruffled exocoelom boundary [44]. Conversely, reduced expression of a component of the extracellular matrix, Fibronectin-1, in CHATO-deficient mice results in expansion of the exocoelomic cavity [45]. This suggests that rearrangements of cell-cell and cell-extracellular matrix contacts may play a role in the formation/maintenance of the exocoelomic cavity and the tissues lining it.

Genetic studies in the mouse often provide valuable information on tissue morphogenesis. In contrast to the many mutants described for e.g. allantois [46] and placenta [47], remarkably few mutants appear relevant for our understanding of amnion formation (Table 1). Severe gastrulation mutants often lack an amniochorionic fold due to a general deficit of (extra)embryonic mesoderm, but some mutants display defects primarily related to amnion formation. Remarkably, many of these seem related directly to Bone morphogenetic protein (Bmp) signaling (in particular the *Bmp2 *and *Smad5 *mutant mice, see below), or putative modulators of Bmp signaling (Amn and Bmp1). *Amnionless *(Amn) mutants develop the most specific defects because they lack an amnion, whereas chorion, yolk sac blood islands, and allantois develop normally [33,48]. Interestingly, embryonic ectoderm growth and differentiation are impaired, as well as the correct establishment of the middle - but not the posterior - region of the primitive streak [49]. *Bmp2 null *embryos have a delayed amnion closure or fail to close the amnion, with a proamniotic duct remaining as late as early somite stages. The heart is formed within the exocoelomic cavity instead of the amniotic cavity [34]. Smad5, an intracellular mediator of Bmp signaling, is also implicated in amnion closure: closure is delayed in *Smad5*-deficient mice [31,32] (Figure 7). This phenotype resembles not surprisingly a milder phenotype of the Bmp2 *null *mice. Moreover, the amnion of *Smad5 *mutants often displays local thickenings that contain ectopic PGC-like cells, haematopoietic and endothelial cells; which could be attributed to ectopic Bmp signaling via non-Smad5 pathways [31,50]. This cell agglomerate cannot be related so far with any other loss-of-function model.

**Table 1:** Mutations affecting amnion formation

Gene	Gene Product	Phenotype	Onset Phenotype	Lethality	Reference
*Amn*	Type I transmembrane protein	Amnion absent while extraembryonic structures like chorion, yolk sac blood islands, and allantois develop normally	E 6.5 - Prestreak, no amniotic fold	E 9.5	[33,48,49]

*Bmp1/Procollagen C-proteinase*	Growth factor/protease	Amnion present but lacks the fold covering the loops of the gut in the umbilical ring region	E 11.5	Perinatal	[6]

*Bmp2*	Growth factor	Amnion has a delayed fusion or fails to fuse; heart is formed within the exocoelomic cavity as a result of lack of amnion fusion	E 7.0 - Late streak, large exocoelomic cavity	E8.5 to E9.5	[34]

*Ds *(Ds mutation)	Not known	Semidominant mutation associated with early amnion rupture or *amniotic band sequence *(ABS)	E 18.5	Viable	[58,59]

*Evi1*	Transcription factor with zinc finger motifs	Amnion filled with fluid; unbalanced amniotic ectoderm and mesoderm	E 7.5 - Neural plate, amnion and chorion segregated	E10.5	[60]

*Fibronectin*	High molecular-weight glycoprotein	Undersized amnion; amniotic cavity with pressure deficit	E 7.5 - Neural plate, amnion and chorion segregated	E9.5 to E10.5	[4]

*FoxF1*	Winged helix transcription Factor	Undersized amnion tightens and restricts embryo growth; ectopic VCAM and receptor α1-integrin expression in amniotic mesoderm	E 7.5 - Neural plate, amnion and chorion segregated	E9.5 to E10.5	[44]

*Ldb1*	LIM domain-binding protein	Undersized amnion leading to constricted embryonic-extraembryonic junction	E 7.5 - Neural plate, amnion and chorion segregated	E9.5 to E10	[61]

*Paxillin*	Focal adhesion molecule	Undersized amnion; amniotic cavity with pressure deficit	E 7.5 - Neural plate, amnion and chorion segregated	E8.5 to E9	[62]

*Smad5*	Bmp signaling intermediate	Delayed fusion of the amnion; Thickenings containing ectopic haematopoietic, endothelial and PGC-like cells	E 7.0 - Late streak/early bud, amnion fusion	E9.5 to E10.5	[31,32,50]

*Flk1	VEGF Receptor	In chimeras, Flk1 *null *cells fail to form blood islands and accumulate in amnion	E 7.5 - Neural plate, chorion and ectoplacental cone fuse	E9.5	[63]

*Gastrulation mutants	not applicable	No mesoderm and amniochorionic fold forms, and consequently no amnion	E 6.5 - Prestreak, no amniotic fold	E7.5	[64]

* The observed amnion defects are considered secondary.

We have provided here a morphological description and an animation of the poorly understood process of amnion formation. Nevertheless, we are still a long way from understanding how the process is regulated at the molecular level. Given the poor documentation of gene expression patterns in the amnion, it is at present also unclear if the amnion itself is differentially patterned in anterior versus posterior or lateral amnion. It is unclear what defines or distinguishes progressively embryonic and amniotic ectoderm, or yolk sac and amniotic mesoderm, at the molecular level. Moreover, little is known about the amnion with respect to its impact on the development of the embryo and its surrounding extraembryonic tissues e.g. allantois, yolk sac and chorion, or vice versa. Does the amnion then function exclusively as a container and filter for the amniotic fluid and as a shock absorber? Or does it also signal actively to the surrounding tissues, and hence influences the patterning of the embryo?

Stem cell-like cells have been reported in the human amnion [16,34,51-53] and recently also in the rat [19]. So far, their origin has been speculative. The origin, presence and potential of an amniotic stem cell-like population may differ in primate and rodent embryos because of the difference in topology between the disc-shaped primate and the cup-shaped rodent embryo, and the differences in developmental origin of amniotic layers [9]. However, if amniotic stem cell-like cells exist in mice, mouse genetic models will be extremely valuable for investigating the developmental origin of these cells, as well as in unravelling the complex cascade of molecular events that lead to the appearance of this cell population. The single amniochorionic fold model and the comprehensive animation reported here provide a new framework to investigate this cell population and to examine complex defects in the amnion of mouse mutants.

## Conclusions

Our histomorphological analysis revealed that only one amniotic fold is present in the mouse embryo, which we rename the "amniochorionic fold" (ACF). The ACF emerges at the posterior side of the egg cylinder and expands laterally around the egg cylinder. Exocoelom formation within this fold seems not to involve apoptosis. Here we show that the ACF and exocoelom do not expand through the anterior side of the embryo. Amnion closure is eccentric and occurs close to the anterior margin of the egg cylinder, which we define as the "anterior separation point" (ASP). The 3D reconstructions of historical sections of E7.5 embryos from Kaufman (1992) confirm the single amniochorionic fold model. This model and the comprehensive animation provide a new framework for interpreting fate-map data, investigating amniotic stem cell populations and complex defects in the amnion of mouse mutants.

## Methods

### Histology

For histological analysis, CD1 embryos were collected between E6.0 and E7.5. All experiments were approved by the ethical commission from Katholieke Universiteit Leuven (097/2008). We used the staging nomenclature of embryos that is described in the Edinburgh Mouse Atlas Project [54]. After overnight fixation in 4% paraformaldehyde in PBS at 4°C, the embryos were washed with saline, dehydrated and stored in 70% ethanol at 4°C. The embryos were further dehydrated and embedded in Technovit 8100 (Heraeus Kulzer), sectioned (transverse sections at 7 μm, longitudinal sections at 4 μm) and stained with 0.05% Neutral Red solution. Serial sections of at least 13 embryos per stage were analysed for Figures 2, 3 and 5.

### Terminal deoxynucleotidyl transferase dUTP nick end labeling (TUNEL) assay

TUNEL assays were performed using the In Situ Cell Death Detection Kit, Fluorescein (Roche). Serial sections of 5 paraffin embedded embryos with an emerging amniochorionic fold were analyzed. Sections were deparaffinised using Xylene (VWR) and rehydrated through an ethanol series to distilled water. Permeabilization was done by incubation with 10 μg ProteinaseK/mL (Invitrogen) in 10 mM Tris/HCl pH 7.4 at 30°C during 20 minutes. TUNEL reactions were performed according to manufacturer's instructions. DAPI (Invitrogen) was used to counterstain nuclei.

### In situ hybridization

Whole mount *in situ *hybridization with an antisense probe for *Eomes *[36] was performed as described elsewhere [55], with minor modifications. The embryos were processed afterwards for plastic embedding and sectioning as described above.

### Immunohistochemistry

Immunohistochemistry (IHC) was performed on 4 μm thick paraffin sections of 4% paraformaldehyde fixed embryos using an automated platform (Ventana Discovery, Ventana Medical Systems). We used a rabbit antibody to Oct3/4 (N19, Santa Cruz).

### 3D reconstruction

Images of the original, serial, longitudinal sections of embryos in the collection used for *The Atlas of Mouse Development *(Kaufman 1992) were obtained and stacked using the methods and software developed for the *Edinburgh Mouse Atlas Project *[56]. The image stacks were sliced at the desired level and orientation to obtain perfectly sagittal and transverse slices using MAPaint [57].

## List of abbreviations

**3D**: three dimensional; **AC**: amniotic cavity; **ACF**: amniochorionic fold; **Al**: allantois; **Al-bud**: allantoic bud; **Am**: amnion; **AmEc**: amniotic ectoderm; **AmM**: amniotic mesoderm; **Amn**: amnionless; **ASP**: anterior separation point; **AVE**: anterior visceral endoderm; **BMP**: bone morphogenetic protein; **Ch**: chorion; **De**: deciduum; **DS**: disorganization mutation; **E7.5**: embryonic day 7.5; **EC**: exocoelomic cavity; **Em**: embryo; **EMAP**: Edinburgh Mouse Atlas Project; **En-frw**: endodermal furrow; **Eomes**: Eomesodermin; **EPC**: ectoplacental cavity; **EP-Cn**: ectoplacental cone; **Evi1**: ecotropic viral integration site 1; **ExEc**: extraembryonic ectoderm; **ExM**: extraembryonic mesoderm; **Flk1**: protein-tyrosine kinase receptor; **FoxF1**: Forkhead box protein F1; **IHC**: immunohistochemistry; **Ldb1**: LIM domain binding 1; **Oct4**: octamer-4; **PAC**: proamniotic cavity; **PBS**: phosphate buffered saline; **PEnd**: parietal endoderm; **Pl**: Placenta; **PS**: primitive streak; **PYS**: parietal yolk sac; **RM**: Reichert's membrane; **Smad5**: mothers against decapentaplegic homolog 5; **TE**: trophectoderm; **TS**: Theiler stage; **UC**: umbilical cord; **VEnd**: visceral endoderm; **VYS**: visceral yolk sac; **YSC**: yolk sac cavity.

## Authors' contributions

PNGP understood the need for revisiting the process of amnion formation, carried out the histological studies, *in situ *hybridization, immunohistochemical stainings, TUNEL assays, designed and generated the animation and other schematic representations. MPD collected the material for Figure 1 and generated the figure. LG performed the 3D reconstructions on the original, serial, longitudinal sections of the embryos from the collection used for *The Atlas of Mouse Development *by Kaufman (1992). DH gave essential critical feedback and support on the study. PNGP, KAL and AZ designed the study, conceived the model and drafted the manuscript. All authors read and approved the submitted manuscript.

## Supplementary Material

Additional file 1 **Amnion formation in the mouse embryo**. The animation is an attempt to visualize amnion and exocoelom formation in the mouse embryo, based on the embryos shown in Figures 2, 3, 5 and 6. Relative dimensions are not to scale. For simplicity, the parietal yolk sac is not depicted in the animation. Click here for file
